# Generalized Term Similarity for Feature Selection in Text Classification Using Quadratic Programming

**DOI:** 10.3390/e22040395

**Published:** 2020-03-30

**Authors:** Hyunki Lim, Dae-Won Kim

**Affiliations:** 1 Image and Media Research Center, Korea Institute of Science and Technology, 5 Hwarang-Ro 14-gil, Seongbuk-Gu, Seoul 02792, Korea; hyunkilim@kist.re.kr; 2 School of Computer Science and Engineering, Chung-Ang University, 221 Heukseok-Dong, Dongjak-Gu, Seoul 06974, Korea

**Keywords:** text categorization, information gain, mutual information, chi-square statistic, quadratic programming

## Abstract

The rapid growth of Internet technologies has led to an enormous increase in the number of electronic documents used worldwide. To organize and manage big data for unstructured documents effectively and efficiently, text categorization has been employed in recent decades. To conduct text categorization tasks, documents are usually represented using the bag-of-words model, owing to its simplicity. In this representation for text classification, feature selection becomes an essential method because all terms in the vocabulary induce enormous feature space corresponding to the documents. In this paper, we propose a new feature selection method that considers term similarity to avoid the selection of redundant terms. Term similarity is measured using a general method such as mutual information, and serves as a second measure in feature selection in addition to term ranking. To consider balance of term ranking and term similarity for feature selection, we use a quadratic programming-based numerical optimization approach. Experimental results demonstrate that considering term similarity is effective and has higher accuracy than conventional methods.

## 1. Introduction

The rapid growth of Internet technologies has led to an enormous increase in the number of electronic documents used worldwide. To organize and manage documents effectively and efficiently, text categorization (TC) has been employed in recent decades. TC assigns text documents to pre-defined topics, categories, or classes, which is an important task in information retrieval [1]. TC has been gaining additional traction in recent years owing to easily-available digitized text such as web pages, e-mails, blogs, social network services, product information or reviews, etc. [2].

To conduct TC tasks, documents are usually represented using the bag-of-words model, because of its simplicity. In this representation, dimensionality is high [3,4] because all terms in the vocabulary are used to construct the feature vectors corresponding to the documents. As a matrix representation, the documents and terms correspond to rows and columns, respectively, and the number of terms may reach tens to hundreds of thousands [5]. While dimensionality may be very high, a large number of terms may not be relevant to the topic, and can be considered as noise. Thus, many researchers have proposed different feature selection methods for TC [6,7,8] to reduce dimensionality, to simplify the feature vectors, and to achieve high accuracy and efficiency.

For TC, conventional feature selection metrics measure the dependency between terms and the topic based on term frequency, such as $\chi^{2}$, mutual information, and information gain, and then rank the terms using the dependency values [9]. However, these approaches may select redundant terms because, in large text documents, similar terms occur, and the metrics give similar scores to similar terms (for example, synonyms). Many recent feature selection methods used for TC are also based on these metrics, and may operate under this restriction. Thus, these redundant terms can impose a limit on the accuracy of TC.

In this paper, we propose a novel term selection method to reduce selection of redundant terms by considering term similarity. Term similarity is measured using a general method, such as $\chi^{2}$, and serves as a second measure in feature selection, in addition to term ranking. Our approach induces independent terms to avoid redundant terms and finds various terms for considering many documents that can cover various subjects. For this goal, the proposed method gives independent terms priority to avoid redundant terms. Thus, the method is not limited to select semantically-related terms. Moreover, to consider balance between term ranking and term similarity for selection of appropriate terms from a global perspective, we use a quadratic programming-based numerical optimization approach. Quadratic programming traditionally has been used to several studies because of usable computational procedure [10,11]. Our objective function is a quadratic function that consists of a quadratic term for term similarity and a linear term for term ranking. We calculate optimal weights for term similarity and ranking using quadratic programming, and select useful terms based on the weights.

## 2. Related Works

There have been studies on dimension reduction, such as random projection, that do not use topic information. For TC, Lin et al. discussed two dimensionality reduction techniques, namely latent semantic indexing and random projection, and proposed a hybrid method combining the two [12]. Bingham et al. presented experimental results using random projection for dimensionality reduction in text document data [13]. Torkkola proposed a feature transform method based on linear discriminant analysis using either random projection or latent semantic indexing [14].

Henceforth, we introduce detailed definitions of three classical feature selection metrics that have been widely used and have achieved satisfactory performance in TC tasks. These metrics are $\chi^{2}$ statistic, information gain, and mutual information. The following definitions are based on [6,15]. $t_{i}$ and $C_{j}$ represent a specific term and a specific category, respectively, and the set of all categories is represented by $C=\{C_{1},\ldots ,C_{m}\}$ where *m* is the number of categories.

- a is the number of documents term in which $t_{i}$ and $C_{j}$ co-occur.
- b is the number of documents term in which $t_{i}$ occurs without $C_{j}$.
- c is the number of documents in which $C_{j}$ occurs without $t_{i}$.
- d is the number of documents in which neither $C_{j}$ or $t_{i}$ occurs.

The $\chi^{2}$ statistic is used to measure the lack of independence between $t_{i}$ and $C_{j}$, and it can be regarded as the $\chi^{2}$ distribution with one degree of freedom. It is defined as

(1) $$\chi^{2}\left(t_{i},C_{j}\right)=\frac{M\times {\left(ad-bc\right)}^{2}}{\left(a+c)\times (b+d)\times (a+b)\times (c+d\right)}$$

where *M* is the total number of documents and can be represented as a+b+c+d. Generally, the category-specific scores of a term can be captured with the average value as

(2) $$\chi_{avg}^{2}\left(t_{i},C\right)=\sum_{k=1}^{m}p\left(C_{k}\right)\chi^{2}\left(t_{i},C_{k}\right)$$

where $p(C_{k})$ can be estimated by $\frac{a+c}{M}$. The maximum value can also be used for the score as  

(3) $$\chi_{max}^{2}\left(t_{i},C\right)=\max_{k=1}^{m}\left\{\chi^{2}\left(t_{i},C_{k}\right)\right\}.$$

Information Gain (IG) was first used as a feature selection measure in a decision tree. In a a typical example of a decision tree, the ID3 algorithm iteratively decides the feature that divides classes well using IG [16]. Supervised feature selection methods such as ID3 can identify different categories. The IG of term $t_{i}$ in multi class text data can be defined as [15]

(4) $$\begin{array}{c} IG\left(t_{i},C\right)=-\sum_{k=1}^{m}p\left(C_{k}\right)\log p\left(C_{k}\right)+p\left(t_{i}\right)\sum_{k=1}^{m}p\left(C_{k}|t_{i}\right)\log p\left(C_{k}|t_{i}\right)\\ +p\left(\bar{t_{i}}\right)\sum_{k=1}^{m}p\left(C_{k}|\bar{t_{i}}\right)\log p\left(C_{k}|\bar{t_{i}}\right). \end{array}$$

In the above definition, $P(t_{i})$, $P(C_{k}|t_{i})$, and $P(C_{k}|\bar{t_{i}})$ correspond to $\frac{a+b}{M}$, $\frac{a}{a+b}$, and $\frac{c}{c+d}$, respectively.

Mutual Information (MI) measures the mutual dependency of two random variables [15], and is defined as

(5) $$MI\left(t_{i},C_{j}\right)=\log\frac{p(t_{i},C_{j})}{p\left(t_{i}\right)p\left(C_{j}\right)}$$

where $p(t_{i},C_{j})$ can be estimated by a/M. In MI, the category-specific scores of a term can also be captured using the average value as

(6) $$MI_{avg}\left(t_{i},C\right)=\sum_{k=1}^{m}p\left(C_{k}\right)MI\left(t_{i},C_{k}\right).$$

To conclude, conventional feature selection methods for text categorization evaluate the importance of $t_{i}$ based on its dependency on categories *C*, and the top-scoring features are used in the categorization process without requiring a special search.

Recently, some feature selection methods have been introduced based on classical methods for TC. Uysal proposed an improved global feature selection scheme (IGFSS) that creates a feature set representing all classes almost equally [17]. In the final step of the method, a common feature selection scheme is modified to obtain a more representative feature set. However, when the dataset is imbalanced, the IGFSS has difficulty in selecting a feature set that represents all classes equally. Tang et al. proposed a feature selection method based on a divergence measure for naive Bayes classification [18]. Moreover, they analyzed the asymptotic properties of the divergence measure relating to Type I and II errors of a Bayesian classifier. However, the method is specialized only for the Bayesian classifier. Javed et al. proposed a two-stage feature selection method that combines conventional feature-ranking and feature search for improved efficiency [19]. In their method, the first stage employs a feature-ranking metric such as IG, and in the second stage, a Markov blanket filter is applied. Wang et al. proposed an approach using Student’s *t*-test to measure the diversity of the distributions of term frequency between a specific category and the entire corpus [6]. Uysal et al. proposed a distinguishing feature selector using a filter-based probabilistic feature selection method [20]. They assumed that an ideal filter should assign high scores to distinctive features while assigning lower scores to irrelevant ones. To achieve their objective, they defined a term as a distinctive term if that term frequently occurs in a single class and does not occur in other classes.

## 3. Proposed Method

Let $f(t_{i},C)$ denote a function of the *i*th feature that represents the dependency between the *i*th term (1≤i≤N) and a specific category *C*. *f* is defined to select informative features for TC, and can be any conventional feature selection metric such as those in Equations (2), (4), or (6). Then, the top *n* features are selected by sorting on the function values. In our earlier studies [21,22], we proposed feature selection methods for a multi-label dataset. In this work, we first applied the method for the TC problem, and then used other conventional feature selection metrics for TC to model a new term selection method.

In the proposed method, a penalty is assigned to similar or redundant terms. $f(t_{i},c)$ such as $\chi^{2}$ used in TC is also used in the proposed method, and we add another penalty function. The penalty for similar terms is calculated based on the dependency among terms similar to $f(t_{i},C)$. To calculate the dependency among terms, we use the same function *f* as $f(t_{i},t_{j})$. Then, for $t_{i}$, we obtain values of $f(t_{i},C)$, and $\left\{f\left(t_{i},t_{j}\right)|1\leq j\leq N,i\neq j\right\}$. To select a term that is not similar to other terms, and simultaneously has a high dependency with category *C*, we can define the score for a term $t_{i}$ as

(7) $$J\left(t_{i}\right)=f\left(t_{i},C\right)-\sum_{i=1,i\neq j}^{N}f\left(t_{i},t_{j}\right).$$

In this score, the first term on the right hand side is the conventional feature selection metric and the second is used to consider the similarity with other terms. To calculate the similarity among terms, we define new categories in the perspective of terms using a, b, c, and d in Section 2 as:

- a is the number of documents in which $t_{i}$ and $t_{j}$ co-occur.
- b is the number of documents in which $t_{i}$ occurs without $t_{j}$.
- c is the number of documents in which $t_{j}$ occurs without $t_{i}$.
- d is the number of documents in which neither $t_{j}$ nor $t_{i}$ occurs.

$f(t_{i},t_{j})$ is used as a generalized similarity function by using newly defined a, b, c, and d, and the function can be specifically chosen, e.g., $\chi^{2}$, information gain, or mutual information. For instance, the similarity between $t_{i}$ and $t_{j}$ can be calculated as

$$\chi^{2}\left(t_{i},t_{j}\right)=\frac{M\times {\left(ad-bc\right)}^{2}}{\left(a+c)\times (b+d)\times (a+b)\times (c+d\right)}.$$

However, all $f(t_{i},t_{j})$ should not be calculated because, when the final term set contains only one of $t_{i}$ or $t_{j}$, then $f(t_{i},t_{j})$ is meaningless. In other words, the score function can be different based on the number of selected terms. For example, if we select three terms, numbered 1, 2, and 3 features from a total of five terms, then we need not calculate $f(t_{1},t_{4})$, … $f(t_{1},t_{5})$. Thus, we should consider the relative importance of the terms; and not the simple score function for a term $t_{i}$.

Let *S* be the final feature subset. Then, we can define the feature selection problem as

(8) $$\max_{S}J=\sum_{t_{i}\in S}f\left(t_{i},C\right)-\sum_{t_{i},t_{j}\in S}f\left(t_{i},t_{j}\right).$$

Although a score function that considers term similarity has been designed, selecting the best feature subset is impractical because the number of feature subset candidates can be $2^{N}$. To circumvent the combinatorial optimization problem, we transform the score function in Equation (8) into a numerical optimization problem, namely quadratic programming.

Let $x\in R^{N}$ be a weight vector and $x_{i}$ be an element that represents the relative importance of the *i*th term. The relative importance of each term is represented as a continuous value between zero and one. The weight vector *x* has the following constraints:

(9) $$x_{1},\ldots ,x_{N}\geq 0,\sum_{i=1}^{N}x_{i}=1.$$

As a result, the score function (8) for the term subset can be transformed to

(10) $$\max_{x}J=\sum_{t_{i}}f\left(t_{i},C\right)x_{i}-\sum_{t_{i},t_{j}}f\left(t_{i},t_{j}\right)x_{i}x_{j}.$$

In the new score function in Equation (10), the combinatorial optimization problem in Equation (8) has been transformed into a numerical optimization problem. Moreover, Equation (10) can be rewritten in the quadratic form as

(11) $$\max_{x}J=c^{T}x-\frac{1}{2}x^{T}Qx$$

where $c\in R^{N}$ is a vector and each element of *c* is defined as

(12) $$c_{i}=f\left(t_{i},C\right),$$

and $Q\in R^{N\times N}$ is a symmetric matrix and each element of which is defined as

(13) $$Q_{i,j}=f\left(t_{i},t_{j}\right).$$

The score function in Equation (11) is now in typical quadratic programming form. If matrix *Q* is a positive definite matrix, then we can obtain the optimal *x* because J(x) is a convex function [23]. In other words, the numerical optimization problem in Equation (11) for TC can now be solved more easily. For the positive definiteness of matrix *Q*, shift eigenvalue correction can be used a solution [24]. The original matrix *Q* is decomposed as

(14) $$Q=U\Lambda U^{T},$$

where *U* and Λ contain the eigenvectors and corresponding eigenvalues of *Q*. Then, the shift eigenvalue correction can be calculated as [25]

(15) $$Q^{*}=QV_{shift}V_{shift}^{T}Q,$$

where $V_{shift}=U{\left|\Lambda \right|}^{-1}{\left(\Lambda -\nu I\right)}^{\frac{1}{2}}$ and ν is the smallest value of Λ. Other techniques for positive definiteness can also be used [24,26].

The steps of the algorithm for the proposed method are as follows;

1. Calculate feature ranking using a common measure such as $\chi^{2}$ for Equation (12).
2. Calculate the dependency among features using the same measure for Equation (13).
3. Solve the optimization problem Equation (11) and select the top *n* features by *x*

Algorithm 1 represents the detailed pseudo-code of the proposed method. On Line 14, to solve the optimization problem, we use the interior point method from the ‘optimization toolbox’ in MATLAB. Ye et al. demonstrated that convex quadratic programming can be done in $O(N^{3})$ arithmetic operations by an iterative algorithm such as the interior point method where *N* is the dimension of *x* [27]. The proposed method consumes time for three parts: calculating $f(t_{i},t_{j})$ and $f(t_{i},C)$, shift eigenvalue correction, and solving quadratic programming. Calculating $f(t_{i},t_{j})$ is the largest part in time consumption. Thus, the time complexity of the proposed method $O(N^{2})$.
**Algorithm 1** Pseudo-Code of the Proposed Method.1:**Input:**2:     $T=\{t_{1},\cdots ,t_{N}\}$, $C=\{C_{1},\cdots ,C_{m}\}$, *n*; $t_{i}$ and $C_{j}$ are the *i*th term and *j*th topic of documents, respectively, and *n* is the number of terms to be selected3:**Output:**4:     *S*; where *S* is the final subset with *n* terms5:**Process:**6:     initialize $Q\in R^{N\times N},c\in R^{N}$7:     **for all** i=1 to *N*8:          $c_{i}\leftarrow f\left(t_{i},C\right)$ using one among Equations (2), (3), (4), and (6)9:          **for all** j=i+1 to *N*10:              $Q_{i,j}\leftarrow f\left(t_{i},t_{j}\right)$ using one among Equations (2), (3), (4), and (6)11:          **end for**12:     **end for**13:     $Q\leftarrow Q+Q^{T}$14:     Calculate eigenvectors *U* and corresponding eigenvalues Λ of *Q*15:     v← the least value of Λ16:     $Q^{*}\leftarrow QV_{shift}V_{shift}^{T}Q$ where $V_{shift}=U{\left|\Lambda \right|}^{-1}{\left(\Lambda -vI\right)}^{1/2}$17:     Solve the problem $\max_{x}\left[c^{x}-\frac{1}{2}x^{T}Q^{*}x\right]$ with constraints to $\sum_{i}x_{i}=1$ and $x_{i}\geq 0$18:     Rank the terms with descending order of *x* and select the top *n* terms

## 4. Experimental Results

### 4.1. Experimental Setup

To validate the performance of the proposed method, we conducted experiments on three datasets: 20-Newsgroups (20NG), Reuters, and Topic Detection and Tracking (TDT). These three datasets have been widely used in TC research for performance evaluation. The 20NG dataset consists of approximately 20,000 documents collected from the postings of 20 different online newsgroups, and the number of categories is relatively balanced. The Reuters dataset originally consisted of approximately 20,000 documents and 135 topics. However, some documents belong to multiple topics and the the distribution among topics is imbalanced. Following the work of Mccallum et al. [28], the Reuters dataset was separated into Reuters10 or Reuters20, consisting of the documents of the first 10 and first 20 topics, respectively. We used Reuters10 in our experiments. It consists of 7285 documents, and each document contains 48.6 terms on average. The TDT dataset consists of approximately 10,000 documents from newswires, radio programs, and television programs [18]. The documents of the TDT dataset also have multiple and imbalanced topics. We used the first 10 topics with the highest number of documents in our experiments, calling the dataset TDT10. The TDT10 dataset consists of 7456 documents, and each document contains 174.1 terms on average. Table 1 shows detailed information about the text datasets.

We used the $F_{1}$ measure to evaluate the classification performance. The $F_{1}$ measure is one of the most popular measure, and is defined as

(16) $$F_{1}=\frac{2\times p\times r}{p+r}.$$

Precision (*p*) is the percentage of documents that are correctly classified as positive from the documents that are classified as positive, and recall (*r*) is the percentage of documents that are correctly classified as positive from all documents that are actually positive. The metrics are defined as

(17) $$\begin{array}{c} p=\frac{TP}{TP+FP}\\ r=\frac{TP}{TP+FN}, \end{array}$$

where TP denotes the number of true positives, FP denotes the number of false positives, and FN denotes the number of false negatives. For multi-category TC, $F_{1}$ is used in two ways, i.e., the micro-$F_{1}$ and macro-$F_{1}$ as

(18) $$\mathrm{micro}-F_{1}=\frac{\sum_{i=1}^{m}F_{1}\left(i\right)}{m}$$

(19) $$\mathrm{macro}-F_{1}=\frac{2\bar{p}\times \bar{r}}{\bar{p}+\bar{r}}$$

where $F_{1}\left(i\right)$ is the $F_{1}$ value of the predicted *i*th category, and $\bar{p}$ and $\bar{r}$ are the precision and recall values across all categories, respectively.

We used the naive Bayes classifier to classify with multinomial distribution, and obtained the classification results with 100, 120, 140, …, 1000 features that were selected by feature selection methods. To demonstrate the superiority of the proposed method, we compared the proposed method with two types of methods. First, we compared the conventional feature selection metrics, $\chi_{avg}^{2}$, $\chi_{max}^{2}$, IG, and MI${}_{avg}$, with the proposed method. Second, we compared the recent feature selection methods for TC with the proposed method. The methods are IGFSS [17], *t*-test [6], and the Distinguishing Feature Selector (DFS) [20].

### 4.2. Comparison Results

**Figure 1:**
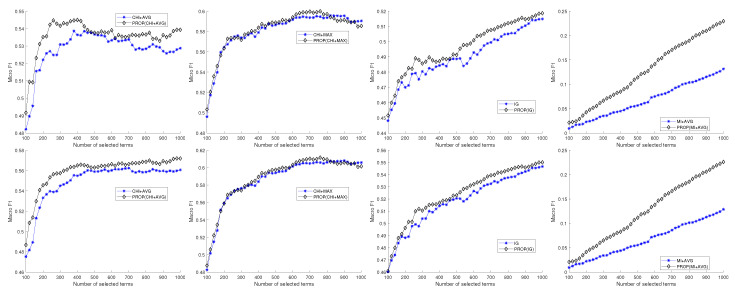
Experimental comparison result of naive Bayes classifier for 20NG dataset.

Figure 1 shows the four comparison results, $\chi_{avg}^{2}$, $\chi_{max}^{2}$, IG, and MI${}_{avg}$, for the 20NG dataset. Upper and lower figures represent micro- and macro-$F_{1}$ results, respectively. In $\chi_{MAX}^{2}$ results (the figures of the second column), the proposed method shows results similar to the original feature selection method. However, the best performance is obtained in the proposed method when the number of selected features is about 700. The other results show that the proposed method outperforms original feature selection methods regardless of the number of selected features. Most results show that $F_{1}$ performance increases steeply before the number of features is 300, and then increase slowly. The MI${}_{avg}$ result shows the lowest performance.

**Figure 2:**
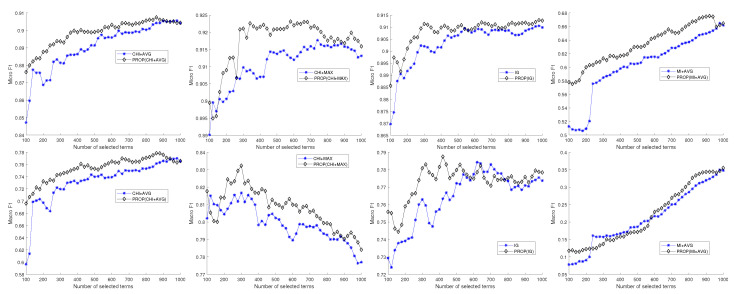
Experimental comparison result of naive Bayes classifier for Reuters10 dataset.

**Figure 3:**
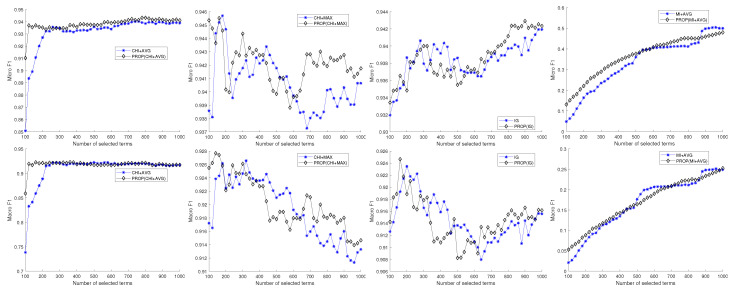
Experimental comparison result of naive Bayes classifier for TDT10 dataset.

Figure 2 shows the results for the Reuters10 dataset. The four subfigures show tendencies similar to those in Figure 1 corresponding to the 20NG dataset. Overall, the results show that the performance of the proposed method is better than that of other feature selection methods. Figure 3 shows the results for the TDT10 dataset. The oscillations over the number of selected features occur because the $F_{1}$ measure is bounded within a small range from 0.91 to 0.92. From the results in these figures, we can conclude that considering term similarity can be an effective mechanism for TC.

**Figure 4:**
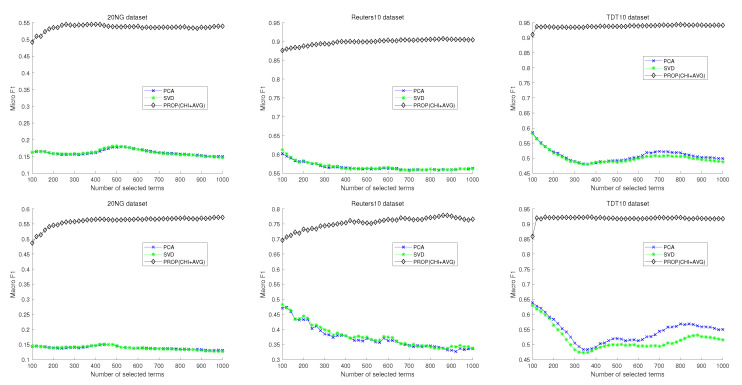
Experimental comparison result of naive Bayes classifier with conventional feature transform methods and the proposed method.

We compared the proposed method with latent semantic indexing based on Principle Component Analysis (PCA) or Singular Value Decomposition (SVD) because feature transform methods are widely used in TC [29]. In this case, the proposed method is designed based on the $\chi_{max}^{2}$ method. Figure 4 shows the results of comparison of the proposed method with conventional feature transform methods. The three subfigures in Figure 4 correspond to the 20NG, Reuters10, and TDT10 datasets, respectively. Upper and lower figures represent micro- and macro-$F_{1}$ results, respectively. In all cases, the proposed method outperforms feature transformation methods. In most cases, when the number of transformed feature is larger, feature transform method is getting worse. Due to nature of transform method that finds largest variance, many features can aggravate performance. However, the proposed method is stable when the number of terms is larger because of balance of term ranking and term similarity.

**Figure 5:**
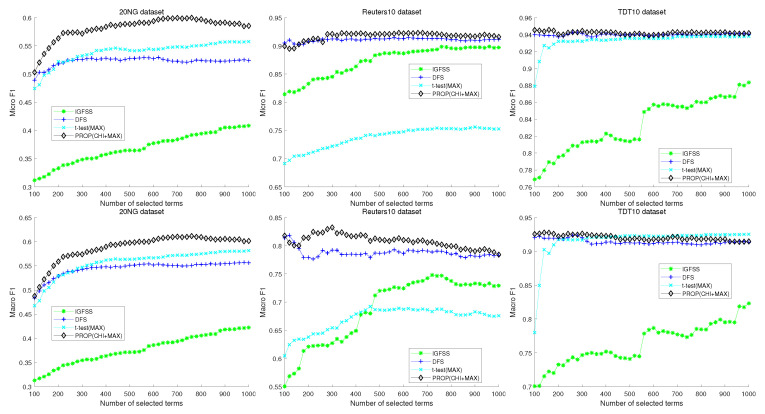
Experimental comparison result of naive Bayes classifier with conventional feature selection method.

Figure 5 shows the results of comparison of the proposed method with more recent feature selection methods. In this case, the proposed method is also designed based on the $\chi_{max}^{2}$ method. The three subfigures in Figure 5 correspond to the 20NG, Reuters10, and TDT10 datasets, respectively. Upper and lower figures represent micro and macro-$F_{1}$ results, respectively. In the case of the 20NG dataset, the proposed method shows better micro- and macro-$F_{1}$ measures than other methods regardless of the number of selected features. The second figure shows the result of Reuters10. When the number of selected features is more than 300, the proposed method shows the best performance. In the TDT10 dataset, the proposed method shows better micro-$F_{1}$ measure than the other methods regardless of the number of selected features. In macro-$F_{1}$ measure, the proposed method and DFS show very similar performance. However, the best performance is obtained in the proposed method when the number of selected features is 160.

Table 2 and Table 3 show the experimental results of each comparison method, when the number of selected terms is 300. To obtain statistically meaningful result, we conducted a holdout cross-validation; 70% of the documents in a given dataset were randomly chosen as the training set, and the remaining 30% of the documents were used as the test set. Each experiment was repeated 30 times, and the average value was used to represent the classification performance according to each conventional method. The best performance among the four comparison methods is represented in bold. † indicates that the proposed method is statistically superior to all conventional methods based on the paired *t*-test (0.05 significance level). In all cases, the proposed method shows better performance than the conventional methods. In most cases, the proposed method shows statistically superior performance.

### 4.3. Analysis of the Proposed Method

In this subsection, we analyze the proposed method with Type I and II errors, and the execution time. Type I and II errors are terms used in statistical hypothesis testing. A Type I error is the incorrect rejection of a true null hypothesis, while a Type II error is the incorrect retention of a false alternative hypothesis. In text categorization, Types I and II correspond to false positives and false negatives, respectively. Table 4, Table 5 and Table 6 show Type I and II errors with 500 selected features and 10 topics for the proposed method. In the 20NG dataset, the Type I error is nearly equal to the number of true positives while the Type II error is very small compared to the number of true negatives. In the Reuters10 and TDT10 datasets, Type I and II errors are much smaller than the numbers of true positives and negatives. These results verify that the selected features of the proposed method reduce the classification error.

We ran experiments in the MATLAB environment with an Intel Xeon processor and 16 GB memory. The proposed method requires 143, 12, and 86 min to complete for 20NG, Reuters10, and TDT10 datasets, respectively. Owing to term similarity evaluation, the proposed method takes more time than the classical feature selection methods such as $\chi^{2}$ statistics. If a low-rank approximation technique such as Nyström method is used for the *Q* matrix, then time consumption can be reduced [30]. Reducing the time complexity can be considered as an area of future work.

## 5. Conclusions

We present the potential of using term similarity when selecting features for TC. Experimental results show that the proposed method outperforms conventional feature selection methods. The proposed method considers not only the dependencies between terms and topics, but also the dependencies among terms. Furthermore, the proposed method finds the optimal balance between two dependencies for feature selection using a numerical optimization approach. We can conclude that considering term similarity reduces the number of redundant terms selected and improves categorization accuracy.

Despite the simplicity and superiority of the proposed method, it suffers from high processing time requirements. Compared with simple conventional methods, the proposed method considers more dependencies among terms, and this increases the processing time. Our future work will include the study of methods to address this limitation.

## Figures and Tables

**Table 1:** Datasets used in the experiments.

Datasets	Documents	Terms	Topics	Average Terms	Maximum Terms
				in Each Document	in a Document
20NG	18,774	11,745	20	131.6	6216
Reuters10	7285	5204	10	48.57	464
TDT10	7456	12,867	10	174.1	1392

**Table 2:** Experimental micro-$F_{1}$ results of naive Bayes classifier when the number of selected terms is 300.

Datasets	IGFSS [17]	DFS [20]	t-test (MAX) [6]	Proposed
20NG	0.3757	0.5505	0.1901	**0.5880** ${}^{†}$
Reuters10	0.8246	0.8904	0.6293	**0.8920** ${}^{†}$
TDT10	0.8103	0.9411	0.3576	**0.9419** ${}^{†}$

**Table 3:** Experimental macro-$F_{1}$ results of naive Bayes classifier when the number of selected terms is 300.

Datasets	IGFSS [17]	DFS [20]	t-test (MAX) [6]	Proposed
20NG	0.3846	0.5665	0.1825	**0.5880** ${}^{†}$
Reuters10	0.6220	0.7696	0.3085	**0.7794** ${}^{†}$
TDT10	0.7502	0.9271	0.3122	**0.9278**

**Table 4:** Type I and II errors of the proposed method in 20NG dataset.

Topic Index	1	2	3	4	5	6	7	8	9	10
Type I error	211	346	371	344	184	192	227	106	80	123
Type II error	112	178	101	142	106	122	109	124	51	70
True Positive	206	211	290	250	277	268	273	271	346	327
True Negative	6770	6743	6769	6938	6923	6896	7004	7028	6985	6988

**Table 5:** Type I and II errors of the proposed method in Reuters10 dataset.

Topic Index	1	2	3	4	5	6	7	8	9	10
Type I error	15	31	34	64	76	67	26	32	35	25
Type II error	25	17	6	1	4	3	4	1	1	0
True Positive	1015	603	92	72	65	54	31	23	20	20
True Negative	1002	1406	1925	1920	1912	1933	1996	2001	2001	2012

**Table 6:** Type I and II errors of the proposed method in TDT10 dataset.

Topic Index	1	2	3	4	5	6	7	8	9	10
Type I error	73	56	5	3	2	1	65	19	1	7
Type II error	27	7	3	0	0	1	1	0	0	0
True Positive	529	572	348	239	132	127	85	67	66	53
True Negative	1628	1602	1881	1995	2103	2108	2086	2151	2170	2177
